# Construction of a predictive nomogram for functional recovery after Bernese periacetabular osteotomy

**DOI:** 10.3389/fsurg.2024.1343823

**Published:** 2024-07-26

**Authors:** Panzhihao Du, Yange Gu, Wenshu Jin, Shufeng Li, Yaohui Yue, Huaqiang Sun, Xinfeng Yan

**Affiliations:** ^1^ Shandong First Medical University & Shandong Academy Medical Sciences, Jinan, Shandong, China; ^2^ Department of Orthopedic Surgery, The First Affiliated Hospital of Shandong First Medical University & Shandong Provincial Qianfoshan Hospital, Shandong Key Laboratory of Rheumatic Disease and Translational Medicine, Jinan, Shandong, China; ^3^ Liver Transplantation Center, General Surgery, Huashan Hospital, Fudan University, Shanghai, China

**Keywords:** developmental dysplasia of the hip, acetabular dysplasia, Bernese periacetabular osteotomy, nomogram, orthopedics

## Abstract

**Background and purpose:**

Surgical indications for Bernese periacetabular osteotomy (PAO) are well-established. However, the extent of postoperative functional recovery varies widely, as observed in clinical follow-ups. Thus, preoperative evaluation is crucial. This study aims to identify factors that influence functional recovery post-PAO and to develop a predictive nomogram.

**Patients and methods:**

Retrospective data were collected between December 2016 and March 2022 at The First Affiliated Hospital of Shandong First Medical University. The dataset included demographic and imaging data of patients who underwent PAO. The least absolute shrinkage and selection operator (LASSO) regression was utilized to identify influencing factors, which were further analyzed using multivariate logistic regression to construct a predictive nomogram for post-PAO functional recovery.

**Result:**

The analysis identified critical factors affecting functional recovery post-PAO, namely, the preoperative distance from the innermost surface of the femoral head to the ilioischial line, the surgical approach, preoperative acetabular depth, and the continuity of the preoperative Calve line. A nomogram was developed using these significant predictors. The model's validity was demonstrated by the receiver operating characteristic curve, with an area under the curve of 0.864. Additionally, the calibration curve confirmed the nomogram's accuracy, showing a strong correlation between observed and predicted probabilities, indicating high predictive accuracy.

**Conclusion:**

This predictive nomogram effectively identifies patients most suitable for PAO, providing valuable guidance for selecting surgical candidates and determining the appropriate surgical approach.

## Introduction

Ganz et al. (1) first described Bernese Periacetabular Osteotomy (PAO) in 1988. Since then, PAO has evolved into a well-established surgical technique for treating developmental dysplasia of the hip (DDH). Studies on post-PAO functional recovery have consistently reported significant pain relief, restoration of hip joint function to normal levels, and marked improvements in patients' daily activities and quality of life (2–5).

Although surgical criteria for PAO are established (6), the extent of postoperative functional recovery varies, as observed in clinical follow-ups. Consequently, preoperative evaluation is essential. Various methods are used to assess the morphology and function of the acetabulum before PAO, including MRI to evaluate labrum and cartilage damage, subchondral cysts, and early signs of osteoarthritis (7); three-dimensional three- dimensional computed tomography to analyze acetabular coverage and version (8–10); and CT scans aligning the hip and knee to measure femoral neck anteversion (11). However, the high cost, ionizing radiation exposure, and the demanding technical requirements limit the widespread use of these diagnostic methods in clinical practice, making them inaccessible for all PAO candidates.

Periacetabular osteotomy is a highly effective hip-preserving surgery, with patients holding high expectations for their surgical outcomes and subsequent quality of life. While some PAO procedures achieve excellent technical success, they may still fall short of meeting patients' preoperative expectations, leading to perceived treatment failures. Often, young patients report mild discomfort as their initial symptom; thus, within the limited treatment timeframe, clinicians must provide a clear DDH diagnosis and straightforward treatment options. This clarity not only facilitates effective doctor-patient communication but also aids in selecting suitable surgical candidates. Although appropriate preoperative evaluations can predict PAO outcomes effectively, a simple, accurate, and practical preoperative standard is lacking. Therefore, we collected demographic data, monitored changes in x-ray parameters pre- and post-surgery, and tracked patients' functional recovery. Using this data, we developed a clinical prediction model to forecast post-PAO recovery by identifying factors that contribute to complete recovery, and subsequently validated its predictive accuracy.

## Patients and methods

### Inclusion and exclusion criteria

This is a single-center retrospective study. The inclusion criteria were: (1) the patient underwent PAO between December 2016 and March 2022; (2) demographic factors, preoperative and postoperative x-rays, and follow-up data were available for analysis. The exclusion criteria were: (1) an exciting nerve, muscle, or connective tissue disease; (2) a history of hip surgery; (3) postoperative imaging parameters were not within the target range of treatment [lateral center-edge angle (LCEA) corrected to 25°–40°, Tonnis angle corrected to 0°–10°] and pelvic posterior column fractures; (4) severe joint deformity; (5) acetabular retroversion (positive cross sign).

### Approach and acetabular displacement

All PAOs were performed using the Modified Smith–Petersen (MSP) or ilioinguinal approaches (12). After completion of the osteotomy, the osteotomy block was clamped by the acetabular reset forceps and rotated to a satisfactory position with the Tonnis angle close to 0° under perspective. The acetabulum was mildly anteverted, and the acetabular rotation center was unchanged or slightly displaced and fixed with 4.5 mm pelvic screws. The site was then flushed and sutured.

### Radiographic assessment

**Figure 1:**
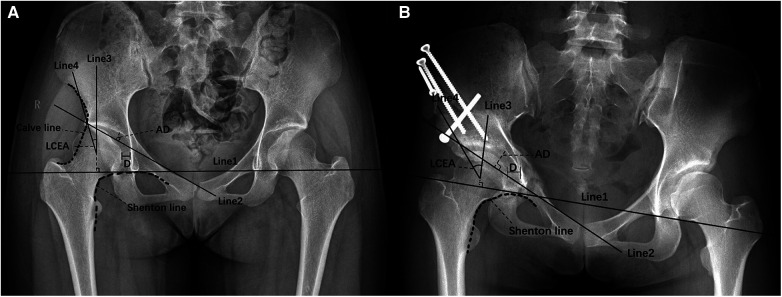
Measurement methods. (**A**) Preoperative pelvic anteroposterior x-ray. Line 1 is the teardrop connection. Line 2 connects the outermost point of the weight-bearing area of acetabular sclerosis and the symphysis pubis's upper corner; the AD is the greatest perpendicular distance from the acetabular roof to Line 2. Line 3 passes through the center of the femoral head and is perpendicular to Line 1, Line 4 is the connection between the center of the femoral head and the outermost point of the weight-bearing area of acetabular sclerosis, and the angle formed by Line 3 and Line 4 is the LCEA. D is the distance from the innermost surface of the femoral head to the ilioischial line. The Calve line is a continuous line between the ilium's outer edge and the femoral neck's outer edge below the anterior inferior iliac spine. The Shenton line is a continuous line between the femoral neck's inner edge and the obturator's upper edge. (**B**) Postoperative pelvic anteroposterior x-ray. Radiographic parameters improved significantly.

An independent observer used standard anterior and posterior x-rays of both hips obtained before the operation and during the clinical follow-up period of at least one year after surgery for radiographic measurements. Radiographic parameters included: the acetabular top tilt angle (Tonnis angle), LCEA, acetabular abduction angle (ABA), femoral head extrusion index (EI), sphericity index of the femoral head, Shenton line, distance from the innermost surface of the femoral head to the ilioischial line, osteoarthritis Tonnis grade, joint congruency, p/a ratio and corresponding acetabular anteversion angle (AAA), Calve line, and acetabular depth (AD). All the above radiographic parameters were measured on the standard anterior and posterior x-rays of both hips. The measurement methods of some radiographic parameters are shown in Figure 1, and the undescribed parameter measurement methods are referred to in the previous literature (13).

### Follow-up and assessment

Patients' modified Harris hip scores (MHHS) were collected before surgery and at least one year after surgery. The MHHS is a principal outcome measure used to assess hip joint function. The MHHS system is comprised of three aspects: pain, function, and functional activities. The score distribution is as follows: 44 points for the pain aspect, 33 points for function, and 14 points for functional activities; the total score of the MHHS is 91 points. The lower the pain level and the better the functional recovery, the higher the score. The full score of MHHS was used as the outcome index to explore the influencing factors of complete recovery after PAO, and a predictive nomogram was constructed.

### Statistical methods

The continuous variables were compared with the Mann–Whitney *U*-test (for non-normally distributed data) or *t*-test (normally distributed data) and expressed as median (interquartile range) [M(IQR)] or mean ± standard deviation ($\bar{X}\pm S$). The categorical data were expressed as percentages (%) and analyzed using the *χ*^2^ test and the Fisher exact test. Least absolute shrinkage and selection operator (LASSO) analysis was used to screen variables from all relevant factors affecting functional recovery after PAO and were included in multivariate logistic regression analysis. The analysis results predicted full recovery of hip joint function (FRHF). R software version 4.2.1 and the LASSO and Elastic-Net Regularized Generalized Linear Models package (R Foundation for Statistical Computing, Vienna, Austria) were used to perform LASSO analysis. Multivariate logistic regression was used to perform multivariate analysis and generate a nomogram based on logistic regression coefficients to predict FRHF in patients. To assess the nomogram's capacity to discriminate, we used the receiver-operating characteristic curve (ROC) and concordance index and evaluated the area under the curve (AUC). Calibration curves were utilized to compare the relationship between the estimated prospects and the factual outcomes. Calibration and discrimination were both assessed by bootstrapping with 1,000 resamples. The nomogram and calibration plot were performed using the Regression Modeling Strategies package from R software, and all other statistical tests were performed using R software. The statistical significance level was set at 0.05.

## Result

### Follow-up

Sixty-seven patients (73 hips) underwent PAO within the target time. A total of 11 hips were excluded, including three patients with a history of hip surgery, four patients lost to follow-up, one patient with intertrochanteric osteotomy, three patients with postoperative imaging parameters corrected outside the target range, and no surgical failure (MHHS score ≤ 70). A final cohort of 57 patients (62 hips) was included, including seven males and fifty females. The average follow-up time was 3.4 years, and the average age was 33.6 ± 9.5 years (ranging from 15 to 52 years). The patients' general information is shown in Table 1.

**Table 1:** The patients’ general information.

Demographic parameters	Value
Number of patients (hips)	57 (62)
Age at surgery ($\bar{X}\pm S$, years)	33.6±9.5, (15–52)
Follow-up time [M(IQR), years]	3.5 (2.3)
Approach [case (%)]	
Ilioinguinal	27 (43.5)
Modified Smith–Petersen	35 (56.5)
Gender [case (%)]	
Male	7 (12.3)
Female	50 (87.7)
Side [case (%)]	
Left	23 (37.1)
Right	39 (62.9)
BMI [M(IQR), Kg/m^2^]	22.8 (4.5)

BMI, body mass index.

### Radiographic improvement and functional evaluation

**Figure 2:**
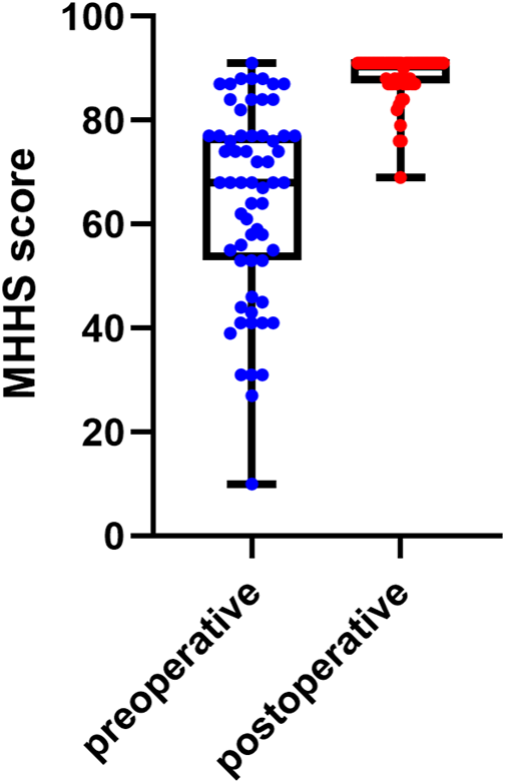
Comparison of preoperative and postoperative modified harris hip score.

The x-ray imaging parameters were significantly improved after surgery (Table 2, Figure 2). The improvement of LCEA, EI, p/a ratio, AAA, AD, ABA, Tonnis angle, hip joint center position and joint congruency were statistically significant. The MHHS was significantly improved (Table 2, Figure 2), and the hip function was improved, with a perfect score of 62.9%.

**Table 2:** X-ray and modified Harris hip score findings of patients before and after surgery.

Radiographic parameters	Preoperative value	Postoperative value	*P*-value
LCEA (M[IQR],°)	10 (8.3)	32 (6)	<0.01
EI ($\bar{X}\pm S$,%)	29.4±8.8	10.1±5.4	<0.01
p/a ratio [M(IQR), %]	2.3 (1.3)	1.9 (0.8)	<0.01
AAA (M[IQR],°)	21 (17.4）	17.9 (7.8)	<0.01
Acetabular Depth ($\bar{X}\pm S$,mm)	6±2.9	14.4±3.8	<0.01
ABA (M[IQR],°)	48 (5.3)	35 (9.8)	<0.01
Tonnis angle (M[IQR],°)	24 (8.6)	5 (6)	<0.01
Hip joint center position ([M(IQR), mm]	11.5 (4.5)	8.9 (3.7)	<0.01
Tonnis grade			0.648
Grade 0	28 (45.2%)	27 (43.5%)	
Grade 1	31 (50%)	33 (53.3%)	
Grade 2	3 (4.8%)	2 (3.2%)	
Grade 3	–	–	
Joint congruency			<0.01
Excellent	30 (48.4%)	51 (82.3%)	
Good	23 (37.1%)	6 (9.7%)	
General	6 (9.7%)	5 (8.1%)	
Poor	3 (4.8%)	–	
Calve line			0.129
Continuous	45 (72.6%)	37 (57.9%)	** **
Discontinuous	17 (27.4%)	25 (40.3%)	** **
Shenton line			0.277
Continuous	46 (74.2%)	51 (82.3%)	<0.01
Discontinuous	16 (25.8%)	11 (17.7%)	
MHHS [M(IQR), score]	68 (24)	91 (4)	

AAA, Acetabular anteversion angle; ABA, acetabular abduction angle; EI, extrusion index; LCEA, lateral center-edge angle; MHHS, modified Harris hip score.

### Preliminary screening of FRHF influencing factors

LASSO regression analysis was used to screen the influencing factors of FRHF after PAO, and lambda was taken as the minimum value. A total of 10 related factors were screened out, which were: side, preoperative ABA, preoperative Tonnis angle, postoperative p/a ratio, postoperative Tonnis grade, preoperative distance from the innermost surface of the femoral head to the ilioischial line, postoperative AAA, surgical approach, preoperative AD, and preoperative continuity of Calve line.

### Nomogram construction

**Figure 3:**
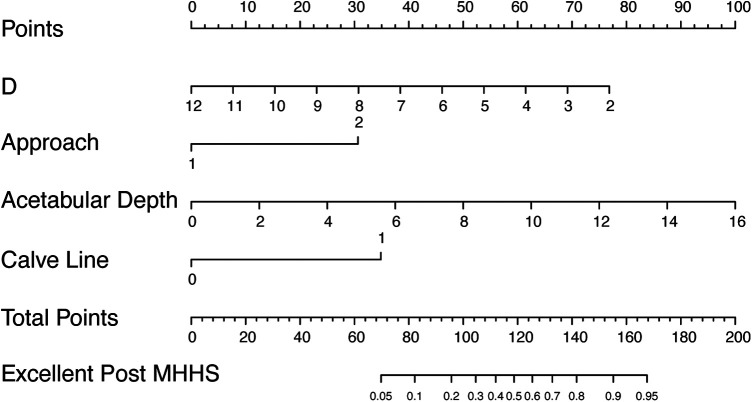
Nomogram for predicting functional recovery after PAO. Codes annotation is as follows: approach, 1 = Ilioinguinal approach, 2 = MSP approach; Calve line, 0 = discontinuous, 1 = continuous; D is the distance from the innermost surface of the femoral head to the ilioischial line.

The selected variables were included in the multivariate logistic regression analysis, and four statistically significant variables were obtained: the distance from the innermost surface of the femoral head to the ilioischial line, surgical approach, preoperative acetabular depth, and preoperative Calve line continuity (Table 3). The nomogram for predicting FRHF is composed of statistically significant variables in the multivariate logistic regression. The nomogram matches the probability of FRHF by accumulating the scores of each risk factor detected on the fractional scale and visualizes this probability on the bottom scale. The higher the score, the greater the probability of achieving FRHF after PAO (Figure 3).

**Table 3:** Results of multivariate logistic regression analysis.

	Estimate	*Z*	*P*-value
Side	2.063	1.879	0.060
Preoperative ABA	−0.034	−0.283	0.777
Preoperative Tonnis angle	−0.224	−1.784	0.074
Postoperative p/a ratio	−0.568	−0.377	0.706
Postoperative Tonnis grade	−1.038	−0.791	0.429
Preoperative hip joint center position	−0.860	−2.282	0.023*
Postoperative AAA	−0.161	−0.945	0.345
Approach	2.816	2.084	0.037*
Preoperative acetabular depth	1.421	2.414	0.016*
Preoperative Calve line	2.659	2.131	0.033*
(Intercept)	10.113	1.167	0.243

AAA, Acetabular anteversion angle; ABA, acetabular abduction angle.

### Evaluation of prediction performance of the nomogram

**Figure 4:**
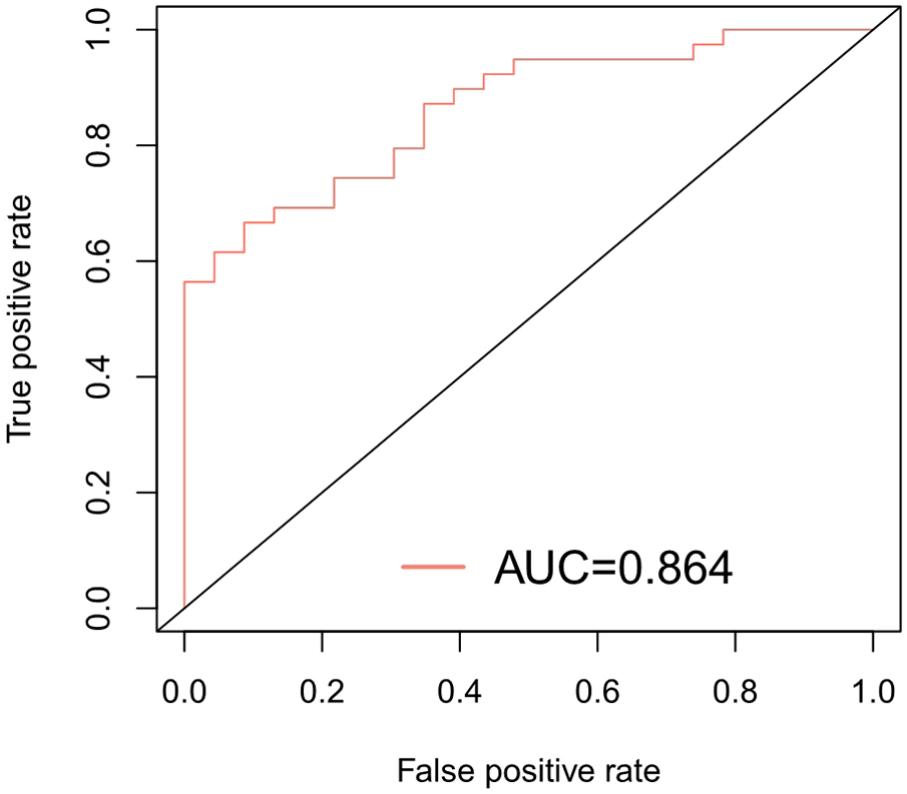
The predictive nomogram's receiver-operated characteristic curve (ROC).

**Figure 5:**
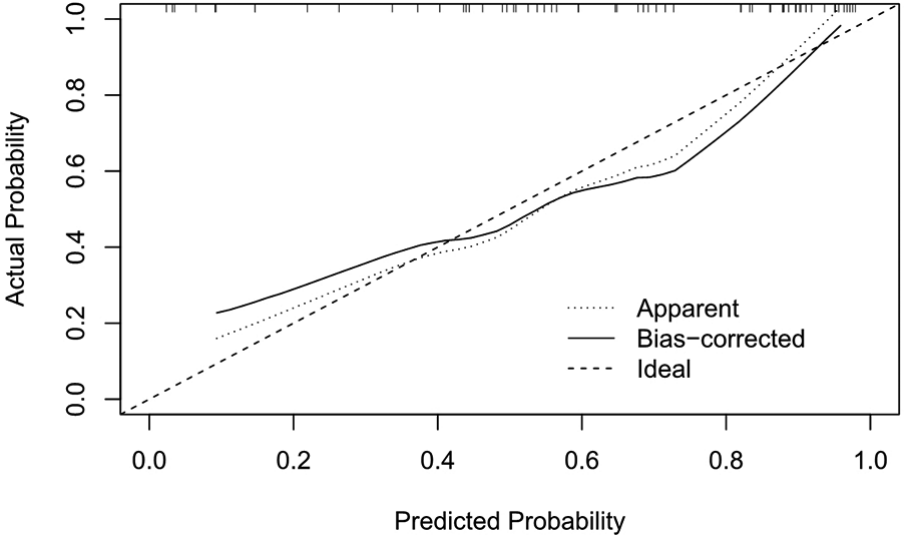
Calibration curve of the predictive nomogram.

The ROC of the model was drawn, and the AUC was calculated to be 0.864 (Figure 4). The calibration curve shows that the constructed nomogram model is well calibrated and that there is sufficient consistency between the observed and estimated prediction probabilities (Figure 5).

## Discussion

The distance from the innermost surface of the femoral head to the ilioischial line indicates the hip center's position, and its lateral shift is a characteristic imaging feature in DDH patients. John C. Clohisy et al. (14) demonstrated that this lateral displacement of the hip center in DDH patients extends the lever arm of gravity and increases joint pressure, potentially causing earlier onset of hip pain and a higher risk of intense pain. The study by Yange Gu et al. (15) showed that the greater distance from the innermost surface of the femoral head to the ilioischial line could lead to early pain symptoms in DDH patients and is a risk factor for severe pain. Our study suggests that the distance from the innermost surface of the femoral head to the ilioischial line clearly affects the functional recovery of patients after PAO, supporting the conclusions of the previous two studies. Therefore, the distance can theoretically guide the assessment of the disease and predict the results of surgery.

The common surgical approaches for PAO are the ilioinguinal and MSP approaches. The MSP approach can provide a good surgical field of vision, facilitate the intraoperative treatment of acetabular fragments, expose the anterior joint capsule, facilitate the opening of the joint and the exposure of the labrum, and treat labrum injury, intra-articular diseases and femoral head and neck deformities. At the same time, it is helpful for neurovascular protection and reduces the risk of neurovascular injury (16–18). However, young female patients may dislike the scar resulting from the local tension of the MSP approach. Studies have shown that the incidence of adverse events after PAO through the ilioinguinal approach is high, including insufficient or excessive osteotomy correction, permanent numbness of the lateral femoral cutaneous nerve, and incisional hernia. Luo et al. (12) compared the outcomes of the MSP and ilioinguinal approaches with the efficacy of PAO. They found no significant difference in the improvement of hip function, but the ilioinguinal approach may have caused more blood loss and required longer surgery. Differing from that study, the surgical approach in our research is one of the factors affecting functional recovery after PAO. Patients who had the MSP approach will benefit more from surgery than those who had the ilioinguinal technique. This finding provides an informed choice for patients faced with the option of different surgical techniques in the future.

Currently, there are many studies on the effect of LCEA on the postoperative recovery of PAO. The hip joint with severe dysplasia has a higher risk of insufficient correction after surgery. Albers et al. (19) conducted a 10-year follow-up after PAO and proved that LCEA < 22° after PAO was an independent risk factor for surgical failure. Based on this study, Novais et al. (20) conducted a study of 128 cases of PAO to study the risk factors of postoperative LCEA < 22°. The results showed that preoperative LCEA was the only factor affecting postoperative LCEA among demographic factors and preoperative radiographic parameters. However, these two studies only screened out a single influencing factor, did not evaluate the efficacy of LCEA, and failed to explain the correlation between preoperative LCEA and postoperative functional recovery. We conducted a retrospective study of 44 PAO patients (13), and the results showed that patients with preoperative LCEA ≥ 4.5° could achieve better functional recovery, which quantified to a certain extent the correlation between preoperative LCEA and postoperative active recovery. Acetabular depth is often used to evaluate acetabular development with LCEA. Previous studies have shown a close correlation between LCEA and acetabular depth in patients with DDH, and LCEA positively correlates with acetabular depth (21). The depth of the acetabulum can be used to compensate for the deviation of the LCEA measurement caused by the hyperplasia of osteophytes. When bone hyperplasia occurs at the outer edge of the acetabulum, which causes the outer edge of the acetabulum to extend outward, the measured LCEA will become larger. When the femoral head appears hypertrophied, and deformation displacement causes the center of the femoral head to move outward, the measured LCEA will be too small. This may be why the acetabular depth and the distance from the innermost surface of the femoral head to the ilioischial line were screened out in this study, but LCEA was not. At present, there is no relevant research on the effect of acetabular depth on PAO postoperative recovery. This research provides a new direction for the study of factors affecting postoperative recovery from PAO.

The discontinuity of Calve line usually indicates hip dislocation or femoral neck fracture, which can indirectly reflect the position of the center of the femoral head and the shape of the acetabulum, thus suggesting the severity of hip dysplasia. In this study, compared with patients with continuous Calve lines, patients with discontinuous Calve lines had poor functional recovery after surgery.

The Tonnis grade of osteoarthritis is one of the influencing factors of functional recovery after PAO. Patients with no or mild osteoarthritis before surgery have less pain and better functional recovery after PAO (22, 23); a Tonnis grade greater than two is a predictor of PAO surgery failure (24, 25). Because only three patients in this study had a preoperative Tonnis grade of two, and the follow-up time was short, the degree of osteoarthritis did not progress significantly, so we could not observe whether the functional score was related to the Tonnis grade. Joel Wells et al. (25) believe that joint matching is also an influencing factor affecting the effect of PAO surgery. Patients with good or excellent joint matching are the best PAO candidates. The joint congruency and other influencing factors were not screened out, which may be due to the small sample size and the slight difference between groups.

We screened a large number of variables that could affect postoperative functional recovery and constructed a predictive nomogram for functional recovery after PAO. Four influencing factors were screened out, and the relevant influencing factors were quantified so that the effectiveness of each could be clearly observed and presented in the form of a nomogram. The model predicts the surgical effect more comprehensively, clearly and accurately. The advantage of this predictive nomogram is that the effect of the operation can be predicted only by x-ray and questioning the patient, saving time and financial cost and reducing the amount of ionizing radiation patients receive. Furthermore, there is no higher requirement for hospitals, so this nomogram can be popularized to most hospitals. During the limited time of outpatient service, the corresponding score can be calculated by x-ray examination to provide patients' expected degree of postoperative functional recovery so that both doctors and patients can make decisions based on the evidence before surgery.

### Limitations

This study had certain limitations. First, our sample size was small. Second, our study was a retrospective case study. This type of study inherently has various sources of bias, including selection bias, measurement and evaluation bias, and loss to follow-up. Third, the radiographic parameters we considered lack femoral parameters, mainly because it is difficult to accurately measure the radiographic parameters of the femoral side only by x-ray. MRI and CT are not routine examinations before PAO, so we did not include femoral parameters. We will incorporate more radiographic parameters in future studies to build a more complete and accurate predictive nomogram.

## Conclusions

The preoperative distance from the innermost surface of the femoral head to the ilioischial line, surgical approach, preoperative acetabular depth, and preoperative Calve line continuity are the influencing factors of functional recovery after PAO. This predictive nomogram effectively identifies patients most suitable for PAO, providing valuable guidance for selecting surgical candidates and determining the appropriate surgical approach.

## Data Availability

The raw data supporting the conclusions of this article will be made available by the authors, without undue reservation.
